# Mesenchymal Stem Cells Transplantation following Partial Hepatectomy: A New Concept to Promote Liver Regeneration—Systematic Review of the Literature Focused on Experimental Studies in Rodent Models

**DOI:** 10.1155/2017/7567958

**Published:** 2017-03-13

**Authors:** Ioannis G. Papanikolaou, Charalambos Katselis, Konstantinos Apostolou, Themistoklis Feretis, Maria Lymperi, Manousos M. Konstadoulakis, Apostolos E. Papalois, George C. Zografos

**Affiliations:** ^1^First Department of Propaedeutic Surgery, “Hippocration” Hospital, University of Athens Medical School, Athens, Greece; ^2^Experimental-Research Center, ELPEN Pharmaceuticals, Pikermi, Attica, Greece

## Abstract

Mesenchymal stem cells (MSCs) are an attractive source for regenerative medicine because they are easily accessible through minimally invasive methods and have the potential to enhance liver regeneration (LG) and improve liver function, following partial hepatectomy (PH) and acute or chronic liver injury. A systematic review of the literature was conducted for articles published up to September 1st, 2016, using the MEDLINE database. The keywords that were used in various combinations were as follows: “Mesenchymal stem cells”, “transplantation”, “stem cells”, “adipose tissue derived stem cells”, “bone marrow-derived stem cells”, “partial hepatectomy”, “acute liver failure”, “chronic liver failure”, “liver fibrosis”, “liver cirrhosis”, “rats”, “mice”, and “liver regeneration”. All introduced keywords were searched for separately in MeSH Database to control relevance and terminological accuracy and validity. A total of 41 articles were identified for potential inclusion and reviewed in detail. After a strict selection process, a total of 28 articles were excluded, leaving 13 articles to form the basis of this systematic review. MSCs transplantation promoted LG and improved liver function. Furthermore, MSCs had the ability to differentiate in hepatocyte-like cells, increase survival, and protect hepatocytes by paracrine mechanisms. MSCs transplantation may provide beneficial effects in the process of LG after PH and acute or chronic liver injury. They may represent a new therapeutic option to treat posthepatectomy acute liver failure.

## 1. Introduction

The global cancer statistics suggest that liver cancer has a high incidence in the globe population, occupying the fifth position among most commonly diagnosed neoplasms worldwide. Despite the scientific progress, liver cancer has high rates of mortality, being, nowadays, the second leading cause of death related to cancer [1]. Epidemiological data from Africa and Asia highlight the increasing number of patients with viral hepatitis, whereas, in Europe and the United States, a significant increase in chronic liver diseases such as alcoholic steatohepatitis or nonalcoholic steatohepatitis is detected, which, consecutively, induce an elevation of liver cancer incidence as well [2]. The prognosis for liver cancer is very poor (overall ratio of mortality to incidence of 0.95), and, as such, the geographical patterns in incidence and mortality are similar. Approximately 75% of all liver cancer arises in Asia, with China accounting for over 50% of the world's burden. According to estimates from GLOBOCAN, the highest incidence rate in the world occurs in Mongolia [2, 3].

Surgery remains the cornerstone in the current treatment of hepatocellular carcinoma (HCC). Nevertheless, unfortunately, the long-term survival rates following hepatectomy for HCC remain unsatisfactory. Major factor which determines these disappointing results is the high rate of postoperative intrahepatic recurrence, although it is unanimous conviction that repeat hepatectomy in the HCC patients has been proved to be the most effective therapeutic strategy for the treatment of recurrent hepatic neoplasms [4–6]. Primary and metastatic malignant liver tumors regularly require extended liver resections, for instance, bi- or trisectionectomy excising more than 70% of the total liver tissue. Moreover, it is well known that, in humans, a 20% to 30% of residual liver tissue is fundamentally required for regeneration. This range of residual liver tissue is in strict dependence on the quality of the hepatocytes and, particularly, their proliferative and metabolic capacities [7, 8].

Mesenchymal stem cells (MSCs) exhibit immunomodulatory properties by attenuating the immune and inflammatory responses by the secretion of cytokines [9, 10]. These features enhanced liver regeneration (LG) in acute and chronic liver pathologies in rodents. Furthermore, MSCs improved LG by presenting proproliferative and antiapoptotic properties, thus hampering disease progress [11–13]. These proregenerative properties of MSCs might also enhance the regenerative capacity of the remaining liver after extensive resection [14].

The aim of the present study was to investigate whether MSCs transplantation promoted regeneration of the remaining liver tissues and improved liver function, in rodent models of partial hepatectomy (PH) and acute or chronic liver injury. They may represent a new therapeutic option to treat posthepatectomy acute liver failure [14, 15].

## 2. Materials and Methods

### 2.1. Study Design

#### 2.1.1. Primary Literature Search

A primary literature search for relevant articles regarding MSCs transplantation following PH was conducted using the MEDLINE database. The articles were published between March 1st, 2004, and September 1st, 2016. A comprehensive search was performed using the following search terms: “Mesenchymal stem cells”, “transplantation”, “stem cells”, “adipose tissue derived stem cells”, “bone marrow-derived stem cells”, “partial hepatectomy”, “liver fibrosis”, “acute hepatic failure”, “chronic hepatic failure”, “cirrhosis”, “rats”, “mice”, “rodents”, and “liver regeneration”. All selected keywords in the primary search are broadly adopted and described in current literature and are included in MEDLINE database, in an extensive body of published studies. Various combinations of the keywords and related terms were used to increase sensitivity. Abstracts from all articles were obtained and those with relevant data on MSCs transplantation following PH were reviewed. Moreover, articles with data on MSCs transplantation in liver fibrosis, acute and chronic liver failure, and liver cirrhosis models were also reviewed. A manual cross-reference search of the bibliographies of relevant articles was conducted to identify studies not found through the computerized search.

**Figure 1:**
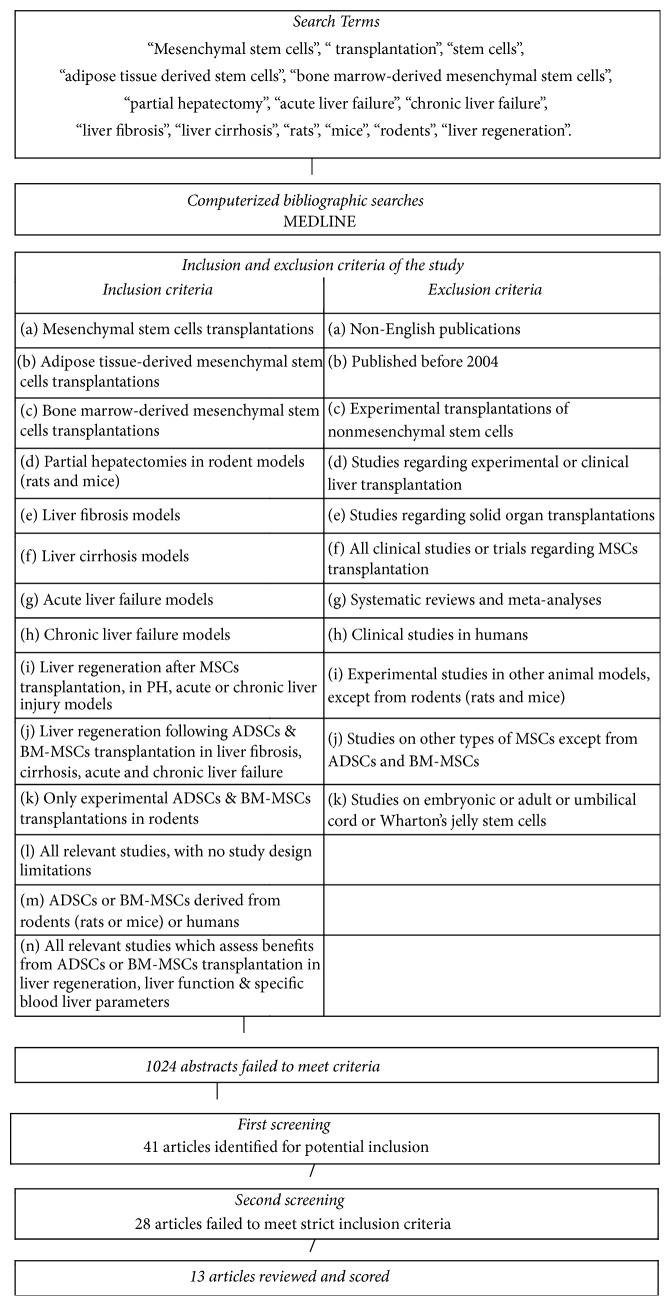
Literature diagram.

Systematic reviews or meta-analyses were not included for methodological reasons. All included studies were experimental studies. No other restrictions were applied on selection. Inclusion and exclusion criteria of this systematic review are reported in Figure 1. All articles which failed to meet strict inclusion criteria were not included in this systematic review.

#### 2.1.2. Secondary Literature Search

A secondary literature search was conducted in the sequel, using Medical Subject Heading (MeSH) terms in MeSH Database, to examine grade of correspondence of primary search terms to MeSH terms. Only a limited number of MeSH terms found in MeSH Database were slightly different from primary search terms. Nevertheless, it should be mentioned that, also in these cases in which terminological discrepancies between primary and secondary literature search were detected, all primary search terms used are thoroughly cited as entry terms in MeSH Database.

Therefore, secondary literature search, served as “a second-level proof” of accuracy and scientific validity, of primary search adopted terminology. Each keyword used in primary literature search was separately searched in MeSH Database, to control terminological accuracy, appropriateness, and validity, using MeSH Database. Primary and secondary literature search terms were compared and had certain terminological discrepancies. Subsequently, a literature search strategy was selected (as described in Section 2.1.3), following strict methodological criteria. Selected search strategy covers the strict spectrum of information needs, researched for the specific aims of this systematic review, and is in accordance with robust methodological design adopted.

In the secondary search, the following Medical Subject Heading (MeSH) terms were retrieved: “Mesenchymal stromal cells”, “transplantation”, “stem cells”, “hepatectomy”, “liver cirrhosis”, “acute liver failure”, “end stage liver disease”, “cirrhosis”, “rats”, “mice”, “rodentia”, and “liver regeneration”.

More specifically, primary search terms “transplantation”, “stem cells”, “acute liver failure”, “cirrhosis”, “rats”, “mice”, and “liver regeneration” were separately searched and are included in MeSH Database, whereas primary literature search term “Mesenchymal stem cells” corresponds to “mesenchymal stromal cells” in MeSH terms. “Mesenchymal stem cells” is a search term, thoroughly cited in MeSH Database, and is retrieved to the entry terms of the MeSH Database. Primary search term “liver fibrosis” stands for “liver cirrhosis” in MeSH Database. However, likewise in this case, primary search term “liver fibrosis” is an entry term of MeSH Database. Furthermore, primary search terms “chronic hepatic failure” and “rodents” correspond to MeSH terms “end stage liver disease” and “rodentia”, respectively. Both primary terms are comprehensively cited in relative entry terms of MeSH Database.

Nevertheless, primary search terms “adipose tissue derived stem cells” and “bone marrow-derived stem cells” were not retrieved in MeSH Database, being explicit, specialized, and specific search terms; however, search results using these primary terms were exhaustively retrieved in MEDLINE. Moreover, primary search term “partial hepatectomy” was not detected as MeSH term, although Medical Subject Heading was found under the generic term “hepatectomy”.

#### 2.1.3. Interpretation of Selected Search Strategy

Considering and comparing primary and secondary search strategy, it was proved that major part of search terms introduced in the primary data search (keywords) coincided with MeSH terms (of the secondary literature search). Nevertheless, terminological inconsistencies between primary and secondary literature search, though insignificant, were, certainly, undeniable. More specifically, as analyzed in previous Section 2.1.2, only a few terms have been slightly different between primary and secondary search and, subsequently, did not coincide terminologically, whereas, even in these cases, it is noteworthy that primary search terms are undoubtedly more established scientific terms, more extensively and assiduously retrievable in published literature, and, subsequently, more intensively and scrupulously adopted by researchers.

As a consequence, primary literature search strategy constitutes main literature strategy for the aims of this systematic review. Secondary literature search served only as a means to control update and accuracy of our selected search terms (keywords), for primary search strategy (which was the main search strategy).

It should be stated that, in all these cases in which primary and secondary search term results did not coincide, primary search terms are thoroughly and comprehensively cited in MeSH Database, as entry terms. It is remarkable fact that entire adopted terminology in primary search is retrieved and scientifically and universally adopted in MEDLINE database, until nowadays, by major part of researchers.

Furthermore, another observation of determinant importance for our research selected strategy was the fact that MeSH terms were found to be more generic, terminologically broad, and imprecise (compared to primary search terms) and, as such, determine detection of misleading, inaccurate results which, evidently, do not correspond to the precise aims and specific literature needs of this systematic review (i.e., “Partial Hepatectomy” versus “Hepatectomy”, “adipose tissue derived stem cells”, and “bone marrow-derived stem cells” versus “Mesenchymal stromal cells”).

This article has a robust design and covers a very specific topic, with strictly defined research and information needs, for which including concrete and restricted keywords is mandatory to exclude methodological confounding factors.

For all the aforementioned reasons, secondary search using MeSH terms was not preferred as main literature search strategy, because MeSH terms could not cover specific information needs of this systematic review.

As a consequence, primary literature search was proved to be strictly affine to our research strategy and was definitely selected as main search strategy, for systematic review specific aims.

#### 2.1.4. Aims of the Study

This article is a systematic review of the literature which aims to collect available evidence on MSCs transplantation following PH and assess its role, in the enhancement of LG and improvement of posthepatectomy liver function, in rodent models. The role of MSCs transplantation will be also examined in other forms of liver injury such as acute and chronic liver failure, liver fibrosis, and liver cirrhosis. It does not fall within the particular aims of this study to investigate specific LG mechanisms, which is a broad topic object of an extensive body of published literature. For that reason, LG will not be specifically looked into or analyzed. LG will be assessed only with particular regard to MSCs transplantation.

### 2.2. Study Endpoints

This systematic review has as primary endpoints investigating outcomes from MSCs transplantation following PH. It examines whether MSCs may enhance the natural LG attended after PH and offer benefits in posthepatectomy liver function.

Secondary endpoints are to assess outcomes from MSCs transplantation in models of acute and chronic liver failure, liver cirrhosis, and liver fibrosis.

A total number of 13 studies were included and reviewed in detail because they followed the strict inclusion criteria of this study. All included and reviewed articles were selected after a strict selection process (Figure 1).

### 2.3. Article Sections

This article has two distinct sections:

1. In the first section, MSCs are defined and classified as follows: adipose tissue-derived mesenchymal stem cells (ADSCs) and bone marrow-derived mesenchymal stem cells (BM-MSCs). Specific regenerative properties of ADSCs and BM-MSCs are investigated, specifically reviewed, and compared. Differentiation protocols of MSCs are referred. Furthermore, mandatory criteria to define MSCs are reported and the stages of ADSCs differentiation into hepatocytes are specifically investigated. In this section, role of ADSCs and BM-MSCs in the enhancement of LG after PH and acute or chronic liver injury is also described. Role of MSCs engineered or in association with scaffold in enhancement of LG is also reported. Engraftment and hepatocyte differentiation of MSCs and profibrogenic potential of human MSC (hMSC) transplant are specifically looked into and compared.
2. In the second section, outcomes from included, eligible experimental studies, after strict selection process, on MSCs transplantation are looked into and discussed. Specific results from ADSCs and BM-MSCs transplantation following PH in rodents are separately reported, discussed, and compared with particular regard to LG and amelioration of liver function. Results from other models of liver injury (acute and chronic liver failure, liver fibrosis, and liver cirrhosis) and subsequent MSCs transplantation are specifically investigated and reviewed.

## 3. Results

### 3.1. Literature Search

Titles and abstracts of 4256 citations were identified from the MEDLINE search engine. After appraisal of the inclusion criteria, 41 articles were identified for potential inclusion and reviewed in detail. A total of 28 articles were excluded for methodological reasons, leaving 13 articles to form the basis of this systematic review (Figure 1). From these articles, 7 studies meet the primary endpoints of this article and are reported in Table 1, whereas 6 studies fulfill the secondary endpoints of the study and are reported in Table 2. All included studies follow the strict inclusion criteria of this systematic review.

### 3.2. Assessment of Included Published Studies

All articles included in this systematic review (*n* = 13), are single-center, experimental studies in rodents (rats or mice). Included studies assess whether MSCs transplantation may represent a means to promote LG and ameliorate liver function. Obtained results are measured qualitatively and not numerically. As a consequence, results of included studies are assessed in a qualitative mode because included published studies have not adopted specific metrics to report obtained LG but only qualitative metrics. This is one of the main weaknesses and shortcomings of published experimental studies which make evident the urgent need of a topical systematic review, which aims to offer high level of evidence.

This is the first systematic review under this specific topic which aims to collect available evidence on “Experimental mesenchymal stem cells transplantation following partial hepatectomy in rodent models”.

### 3.3. First Section

To note is that, in Sections 3.3.7to 3.3.8 of this section, role of MSCs alone on LG is reported whereas, in Sections 3.3.10to 3.3.11, evidence for role of MSCs engineered or in association with scaffold is described, respectively. All mentioned studies, in the first section, do not pertain to eligible and finally reviewed studies for the aims of this systematic review. Reported studies in this section contribute only as further literature proofs for role of MSCs transplantation in LG. Eligible studies which form the basis of this systematic review are specifically looked into, analyzed, and discussed, in the dedicated specific second section (Section 3.4), of this article.

#### 3.3.1. Mesenchymal Stem Cells Regenerative Properties

Defining MSCs and recognizing their properties are essential to conceive their broad therapeutic potential. In a comprehensive effort to define MSCs, it should be stated that MSCs are multipotent and self-renewing cells that are located, substantially in the bone marrow, as a nonhematopoietic cell population, although they may also be isolated from the connective tissues of the major part of organs. MSCs represent a heterogeneous population of adult, fibroblast-like cells which possess the ability to differentiate into tissues of mesodermal lineages including adipocytes, chondrocytes, and osteocytes [28]. In addition to the bone marrow, MSCs have been isolated from various other tissues such as adipose tissue [29], skin [30], heart and spleen [31], placenta [32], and umbilical cord blood [33] as well as lung and liver [34], and it is suggested by published evidence that MSCs reside in the connective tissues of most organs [35].

In recent years, MSCs have been evaluated for their in vivo and in vitro immunomodulatory and “tissue reconstruction” properties, which could make them appealing in various clinical applications and particularly in organ transplantation. Published evidence suggests that intensive in vitro and preclinical research are mandatory to comprehend better the mechanisms with which MSCs act and to attain, in the course of time, the expected clinical success [28].

#### 3.3.2. MSCs Differentiation Protocols

Literature suggests that modulation of MSCs transdifferentiation into hepatocytes is influenced by the following key points: (i) cytokines and growth factors; (ii) cues from the extracellular matrix (ECM); and (iii) physical parameters for MSCs culture. Comprehending the influence of these factors, researchers will be aided to develop improved strategies for inducing the hepatic differentiation of MSCs [36].

Evidence points out that MSCs can be isolated by widening the cell population that vigorously attaches to plastic tissue culture dishes. MSCs are multipotent cells which may give rise to multiple mesenchymal lineages. Numerous published papers indicate that MSCs can also differentiate into hepatocyte-like cells in vitro [37–39].

The differentiation protocols sometimes involve exposure of MSCs to a demethylating drug such as 5-azocytidine pursued by medium that contains typical hepatocytic differentiation factors such as epidermal growth factor (EGF) and hepatocyte growth factor (HGF) [38].

**Figure 2:**
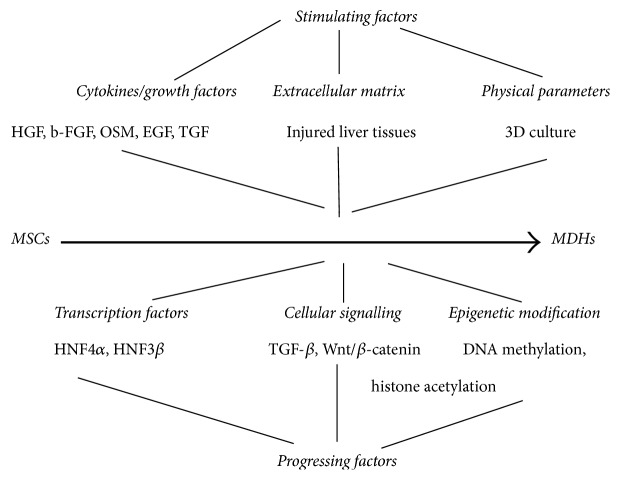
MSCs differentiation into hepatocytes in synthesis. Factors that may induce MSCs differentiation into hepatocytes could be subdivided into 2 subgroups, extracellular (stimulating) factors and intracellular (progressing) factors. Stimulating factors are as follows: cytokines, growth factors, extracellular matrix cues, and physical parameters of culture. Intracellular factors are considered, three key factors, responsible for MSCs differentiation in MSC-derived hepatocytes (MDHs): transcription factors, cellular signalling, and epigenetic modification. MDHs: MSC-derived hepatocytes.

Adipose tissue-derived MSCs have also been induced to evolve into a hepatocyte-like state, accompanied by albumin and glycogen production as well as the ability to detoxify ammonia. This differentiation was induced in culture using a variety of factors such as hepatocyte growth factor (HGF), fibroblast growth factor-1 (FGF-1), fibroblast growth factor-4 (FGF-4), epidermal growth factor (EGF), oncostatin M (OSM), and dexamethasone (Figure 2) [39].

Despite the fact that MSC-derived hepatocyte-like cells have many characteristics of mature liver cells and can engraft in vivo, the extent of functional liver repopulation has, to date, been limited [40].

#### 3.3.3. Why Should ADSCs Be Considered Preferable to BM-MSCs?

Among MSCs, ADSCs are an attractive source for regenerative medicine because they are abundant and can be isolated with minimally invasive procedures [41]. ADSCs are also a convenient source because of their high proliferation and easy maintenance. 1 × 10^8^ ADSCs can be expanded to 10 g of rat epididymal fat pads or human omental fat pads over five passages [42]. Therefore, in the clinical setting, ADSCs could be considered more profitable than BM-MSCs [17].

Furthermore, it has been suggested that ADSCs produce more bioactive factors, including IL-6 and HGF, than do BM-MSCs [43]. There is a growing body of published evidence which suggests the role of ADSCs in increasing LG rate, limiting severity of chronic hepatic failure, and increasing survival [17, 23, 24]. ADSCs can differentiate along multiple cell lineage pathways in a reproducible and regulatable manner. Furthermore, they also can be safely and effectively transplanted to either an autologous or an allogeneic host. It is noteworthy that ADSCs can be manufactured in accordance with current Good Manufacturing Practice guidelines (Table 3) [44].

Meanwhile, BM-MSCs constitute the microenvironment of the bone marrow, regulate hematopoietic function, and possess the ability to differentiate into various cells, including hepatocytes [43–45]. Furthermore, BM-MSCs are easily isolated from bone marrow, readily cultured in vitro, and can be used for autologous transplantation. Thus, they may be ideal seed cells for the treatment of injured tissue. The use of BM-MSCs is also being explored in the fields of regenerative medicine and tissue engineering (Table 3) [20].

#### 3.3.4. Mandatory Criteria to Define MSCs

It should be highlighted that, in regard to MSCs, no specific marker has yet been found. Presently, MSCs are identified using a number of features defined by the International Society for Cellular Therapy which states three minimal criteria [46]: (1) adhesion to plastic in standard culture conditions; (2) expression of CD73, CD90, and CD105 and lack of expression of CD14, CD34, CD45, or CD11b, CD19, or CD79a and Human Leukocyte Antigen- (HLA-) DR surface molecules; (3) in vitro differentiation into adipocytes, chondroblasts, and osteoblasts (Table 3).

#### 3.3.5. Mechanisms Triggering LG Phenomena after MSCs Transplantation

Stem cells may positively influence the recovery from tissue injury via paracrine factors that promote tissue repair [47]. The process of LG after PH, has been reported to initiate in the periportal areas, and the peak of hepatocellular proliferation in this area, is around 24 h after hepatectomy. This knowledge is of extreme importance in triggering LG phenomena [48]. Oval cells are undifferentiated epithelial cells which reside in the periportal area and have been referred to as facultative stem cells [49]. Early production of endothelial IL-6 in the periportal area might be important in the initiation of the LG process [50]. Hepatic stem cells and oval, epithelial, and embryonic cells possess a common marker in the periportal region [51]. As a result those stem cells represent hepatic stem cells reserve. Liver injury affects inflammatory cytokines production. Chemokines are among them and attract BM-MSCs at the site of hepatic injury. BM-MSCs display an even greater role in LG compared to hepatic progenitor cells [52, 53]. In addition, TGF-b shows the ability to dedifferentiate mature hepatocytes and make use of them during cellular expansion of LG [54]. Consequently, it seems to have fundamental importance to define whether MSCs of different origin could further promote the ability of the liver to recover from a serious injury (surgical or chemical) [55].

Certain MSCs features represent favorable parameters in LG. First of all, both types of mesenchymal stem cells, ADSCs and BM-MSCs, express transcription factors responsible for their high proliferative capacity such as Nanog, rex-1, oct-4, and SOX-2 [56]. Also MSCs are capable of differentiating into a wide variety of tissue progenitor cells such as myoblasts, chondroblasts, or hepatoblasts when interacting with a specific microenvironment [56, 57].

As mentioned before, the main advantages of ADSCs are that they are abundant and can be retrieved with minimally invasive procedures [41]. Moreover, it is noteworthy that ADSCs show higher rates of proliferation as well as stronger commitment to hepatic lineage compared to BM-MSCs [56, 58]. Another important characteristic of ADSCs is the fact that they possess cytokine secretory properties. Particularly, ADSCs secrete cytokines such as SCF, GM-CSF, IL-1, IL-6, TGF-b, TNF-a, LIF, VEGF, and PDGF similar to BM-MSCs [59]. A useful property of stem cells is the fact that they have immunomodulatory properties as they can suppress activation of T and B type lymphocytes as well as natural killer cells (NK cells) while, on the other hand, they can be involved in the maturation of dendrite cells [56, 60, 61]. Various studies have investigated the probability of malignant degeneration after MSCs transplantation. This probability is claimed to be quite low, as current evidence has shown [56, 58].

#### 3.3.6. Stages of ADSCs Differentiation into Hepatocytes

Adipose tissue-derived stem cells (ADSCs) represent the most promising candidate progenitor cells for transplantation according to a growing body of evidence in recent years. Indeed ADSCs are located in subcutaneous tissue while distinct surface markers such as CD34^+^ /c-kit^−^, CD90^+^ Thy-1, and, CD105^+^ permit simple accessibility and isolation (compared to BM-MSCs) [52, 53].

In regard to ADSCs commitment rate, which is an issue of crucial importance, ADSCs are committed at higher rates in hepatic lineage (compared to BM-MSCs) after pretreatment with growth factors in order to differentiate into hepatic-like cells [13, 54, 55]. Stimulants such as HGF, EGF, and FGF can influence primary steps of ADSCs differentiation while oncostatin M (OSM) plays a pivotal role in further maturation and expression of hepatocytes properties [56–58, 60]. Finally, a positive feedback loop is described, between growth factors and stem cells as exposure of ADSCs to HGF promotes latter upregulation [61] (Figure 2).

It is noteworthy that pretreatment of ADSCs in vitro induces differentiation which is divided into three discreet stages with complex structural as well as functional changes [54, 59]. As a result cellular shape of stem cells is transformed from spherical into a more polygonal and finally cuboidal shape at the end of maturation, and these events are of fundamental relevance because they prove the differentiation ability of ADSCs in hepatocytic cells [59, 62]. Moreover, it should be mentioned that also E-cadherin expression is upregulated while N-cadherin is downregulated and that shift constitutes the first step in hepatic differentiation. Furthermore, hepatotropic transcription factors (TFs) such as HNF-1, HNF3a/FOXA1, HNF-4a, HNF-6, and GATA4 are activated upon the effect of HGF/c-MET pathway, while Nanog and oct-4 are silenced [63]. Subsequently, proliferative ability of stem cells is halted and specific genes are upregulated (Figure 2). It should be highlighted to that point that hepatic genes production is induced in a timely manner which includes three different stages (Figure 2).

During the* first stage of maturation, from 0 to 8 days, *A-fetoprotein (AFP), cytokeratin 18 (CK18), cytokeratin 19 (CK19), and transthyretin (TTR) show increased levels [28, 64, 65]. During the* second stage, from 9th to 14th day*, albumin (ALB), ornithine transcarbamylase (TAT) as well as mRNA levels for VII clotting factor, asialoglycoprotein receptor 1 (ASGPR1), and CYP1A2 member of cytochrome P450 superfamily production are increased while AFP levels are reduced [65–68]. The* last stage of maturation process, from 15th to 21st day*, presents hepatic-like cells with functional properties unique to hepatocytes such as increased detoxifying ability, lipid and glycogen storage capacity, urea production, and bile acid secretion [65–68].

#### 3.3.7. Evidence from MSCs Transplantation after PH on LG Enhancement

An extensive body of published literature has assessed role of MSCs following PH, in the enhancement of LG. In this direction, Liu et al. [69] transplanted autologous ADSCs and examined obtained LG following a repeat PH in rats. Authors demonstrated that regeneration of remaining liver following R-PH (repeat partial hepatectomy) was significantly promoted by ADSCs transplantation, as proved by a significant increase in liver-to-body weight ratio and the PCNA labeling index at 24 h after hepatectomy. Furthermore, it was detected that the liver essentially fully recovers from hepatocellular damage due to hepatectomy at 168 h postoperatively. These results proved that autologous ADSC transplantation promoted the regenerative capacity of the remnant liver in the early phase following R-PH [69].

Respectively, another research, by Saidi et al. [70], investigated whether human ADSCs attenuate ischemia-reperfusion injury (IRI) and promote LG. In their experiments, mice were subjected to 60 minutes of partial IRI with or without 70% PH. Authors of this study demonstrate that human ADSCs represent a potential therapeutic strategy to decrease IRI and promote LG [70].

Similarly, Chen et al. [71] investigated the role of autologous ADSCs in mice undergoing 70% PH. Authors demonstrated that administration of autologous ADSCs enhances rapid and early LG and, predominantly, preserves function after PH [71].

On the contrary, Liu and Chang transplanted BM-MSCs performing a “wider” PH [72]. In this experimental study, researchers implanted artificial cell bioencapsulated BM-MSCs into the spleens of 90% hepatectomized (PH) rats. The resulting 14-day survival rate was detected to be 91%. This is compared to a survival rate of 21%, in 90% hepatectomized rats, and 25% for those receiving free MSCs transplanted the same way. Notwithstanding free MSCs fate, bioencapsulated MSCs are retained in the spleens and their hepatotropic factors can pursue draining directly into the liver without dilution resulting in improved LG. Moreover, with time, the transdifferentiation of MSCs into hepatocyte-like cells in the spleen renders the spleen as an ectopic liver support [72].

In accordance with other studies, Yu et al. [73] investigated the biological effects of hypoxia-preconditioned BM-MSCs (HP-BM-MSCs) and their utility in a rat model of massive hepatectomy. Authors found that HP-BM-MSCs have improved properties in the setting of hepatocyte proliferation. This functional enhancement of MSCs by hypoxia is not unexpected, as their native environment in the bone marrow is hypoxic and plays an essential role in regulating their function [74]. This research offers a novel approach by which the therapeutic effects of MSCs can be enhanced to improve LG [73].

Another paper by Boeykens et al. [75] investigated the effect of BM-MSCs administration on liver function following PH in rodents. However, despite recovery to normal body weight, liver weight, and NAS score, both serum CHE and TG levels of nontreated and cell-treated rats with PH remained significantly lower as compared to those of control rats. Importantly, serum CHE levels, but not TG levels, of cell-treated rats remained significantly lower as compared to those of nontreated rats.

Further evidence was provided with the study by Kaibori et al. [76], which investigated whether mouse BM-MSCs stimulate LG after PH. Researchers performed a 70% hepatectomy in mice followed by injection of BM-MSCs into the portal vein, or the tail vein, or of saline into the portal vein. Results demonstrated that BM-MSCs injection into the portal vein enhanced LG after PH in mice [76].

In another experimental study Kanazawa et al. [77] investigated the impact of BM-MSCs against hepatic I/R injury and hepatectomy. Authors adopted a new rat model in which major hepatectomy with I/R injury was performed. Remnant LG rate was accelerated in the MSC group. Results of this study suggest that BM-MSCs transplantation provides trophic support to the I/R-injured liver by hindering hepatocellular apoptosis and by enhancing LG [77].

In a recent paper by Okay et al. [78], authors examined the effect of preoperatively administered resveratrol (RV) and MSCs on regeneration of partially hepatectomized rat liver. The effect of RV on homing of MSCs was also assessed. In this study, MSCs were isolated from bone marrow and cultured in vitro. Administration of RV and MSCs, separately or together, enhanced LG despite diminishing the TNF-*α* and IL-6 expression. In addition, RV augmented the homing of MSCs in liver probably related to life prolonging effect on MSCs. These results indicate that preoperative RV as well as MSCs application enhances LG after PH in rats [78].

In their study Adas et al. [79] determined the effects of MSCs therapy and a combination therapy of MSCs transfected with VEGF, for LG, after major resection. Transplanted stem cells and VEGF-transfected MSCs into the portal vein were engrafted in the liver and significantly accelerated many parameters of the healing process following major hepatic resection. Results suggest that MSCs transplantation supported liver function, regeneration, and liver volume/weight [79].

A recent paper by Fouraschen et al. [80] investigated whether liver-derived MSC-secreted factors promote LG after resection in the presence of IRI. In the IRI and PH model, significant reduction in hepatic tissue damage as well as a significant increase in hepatocyte proliferation was observed after MSC-CM (MSC-conditioned medium) treatment. MSC cell-derived factors promote LG of small-for-size livers exposed to ischemic conditions but do not protect against early ischemia and reperfusion injury itself [80].

In a previous study, Fouraschen et al. [81] investigated the effect of human liver-derived MSC-secreted factors in an experimental liver resection model in mice subjected to a 70% PH and treated with either concentrated MSC-conditioned culture medium (MSC-CM) or vehicle control. LG was significantly stimulated by MSC-CM as shown by an increase in liver-to-body weight ratio and hepatocyte proliferation. This study proves that treatment with MSC-derived factors can promote hepatocyte proliferation and LG responses in the early phase after surgical resection [81].

Further evidence by Khuu et al. [82] investigates the in vivo behavior of nondifferentiated adult liver mesenchymal stem/progenitor cells (ADHLSCs) after intrasplenic transplantation into immunodeficient (SCID) mice. Transplanted ADHLSCs were able to differentiate in the nonpreconditioned SCID mouse liver mainly in the periportal area. In response to PH, integrated ADHLSCs proliferate and cooperate with recipient mouse LG [82].

#### 3.3.8. LG after MSCs Transplantation in Rodent Models of Acute and Chronic Liver Injury

Many experimental studies investigate the effects of MSCs transplantation in the enhancement of LG, in models of liver injury. ADSCs transplanted intraportally, rather than through the tail vein, inhibited the proliferation and activation of hepatic stellate cells in vitro and ameliorated liver fibrosis in CCl4-treated rats by improving the microcirculation of the fibrotic liver [83, 84].

In contrast to the similar hepatic integration between undifferentiated ADSCs and ADSCs predifferentiated to hepatocyte-like cells (HLCs) shown in acute liver injury models [85], other liver injury models suggest that predifferentiation of ADSCs to HLCs may facilitate liver engraftment.

In xenogeneic transplantation model of LG, long-term engraftment of human ADSC-derived HLCs was demonstrated and was significantly improved when in vitro predifferentiated ADSCs, instead of undifferentiated MSCs, were used, reaching repopulation rates of more than 10% along with functional LG [37].

In another study, Gruttadauria et al. [86] transplanted BM-MSCs which had not undergone pretreatment with growth factors. As a result stem cells could attenuate ALI and transduce hepatic recovery either incubated or not with hepatogenic mediators [86].

Many researchers tried to investigate the role of MSCs administration in chronic liver injury. MSCs have also been tested to reduce fibroinflammatory reactions, and several studies reported that MSCs inhibit the progression of liver fibrosis. Fang et al. injected BM-MSCs in a mice model of CCl-4-induced liver fibrosis and observed reduced hydroxyproline levels in the serum and fewer histological signs of hepatic necrosis when compared to controls [87].

Other authors utilized infused BM-MSCs, which engraft into host liver and ameliorate fibrosis in a time dependent manner by decreasing *α*-smooth muscle actin expression, reducing collagen deposition, and improving damaged hepatocytes in animal models of experimental liver fibrosis [87–89].

Findings of another study by Oyagi et al. [90] demonstrate that transplantation of ex vivo expanded and HGF-treated BM-MSCs was able to significantly reduce experimental chronic liver injury and fibrosis, supporting the concept that this procedure may be of therapeutic efficacy.

In vitro predifferentiated hepatocyte-like cells were successfully used to treat experimental liver fibrosis. In these studies authors reported that MSCs predifferentiated into hepatocyte-like cells were more efficient for preventing liver fibrosis [91, 92]. MSCs could exert their antifibrotic effects through secretion of matrix metalloproteinases (MMP-9, MMP-13). These enzymes are normally upregulated during liver fibrosis in response to collagen accumulation, and an increase in their activity could allow a more efficient degradation of extracellular matrix [93–95].

Transplantation of different origin MSCs rescued acute liver failure and repopulated mouse liver through paracrine effects that reduced the inflammatory response, inhibited apoptosis in the liver, and stimulated endogenous regeneration mechanisms [96].

In a rat model of hepatic ischemia-reperfusion injury, it has been observed that rat MSCs inhibited hepatocellular apoptosis and stimulated regeneration suggesting a favorable effect of MSCs during acute liver injury [77].

#### 3.3.9. Literature Describing No Benefit of MSCs Transplantation on LG Enhancement

Major part of published evidence on MSCs transplantation suggests their favorable effects in the enhancement of LG and improvement of liver function (Sections 3.3.7 and 3.3.8). Nevertheless, there is published evidence which describes that they have no benefit (reported in this section) and studies which prove their harmful effects (described in Section 3.3.13).

Carvalho et al. observed unchanged transaminase levels, albumin levels, and liver fibrosis area between rats transplanted with BM-MSCs and controls [97]. Bone marrow multipotent mesenchymal stromal cells did not reduce fibrosis or improve function in their rat model of severe chronic liver injury [97].

Further studies in irradiated mice transplanted with sex mismatched bone marrow demonstrated that, in the cirrhotic liver, the contribution of bone marrow to parenchymal regeneration was minor (0.6% of hepatocytes), and, by contrast, the bone marrow contributed to 68% of the hepatic stellate cell pool and 70% of myofibroblast populations [98].

#### 3.3.10. Engineered MSCs Transplantation and Role in LG

In several studies authors transplanted engineered MSCs. To that direction, Itaba et al. [99] manufactured hepatic cell sheets derived from MSCs for treatment of liver failure because cell sheet engineering enabled tissues to retain hepatic functions compared to isolated cell transplantation [100]. Authors examined the therapeutic effects of hepatic cell sheets for acute liver injury in mice. Results of their study reveal that orthotopic transplantation of hepatic cell sheets accelerated LG in mice. Furthermore, this research demonstrated that human MSC-engineered hepatic cell sheets have therapeutic effects on ALI as well as preventive effects. Since these cell sheets technologies have advantages of rapid fabrication and high vascularization, in combination with these excellent technologies, human BM-MSC-engineered hepatic cell sheets hopefully will become promising therapy for liver disease [99].

A therapeutic approach using a combination of MSCs and hepatocytes as a composite liver-assisted device for treatment of rat acute liver failure has previously been reported to provide a long-term survival benefit [101].

More recently, very interesting work by Takebe et al. suggested the importance of MSCs for natural liver development [102].

On this basis, Kadota et al. [103] hypothesized that MSCs would be strong candidates for supportive cells in transplantable whole-organ engineered grafts, which could result in long-term well-maintained hepatocyte function. Authors demonstrated that BM-MSCs are beneficial as supportive cells for the functional efficacy of this engineered liver graft. Authors of this study have documented the positive effects of MSCs on LG. Results of this research support the notion that the angiogenic potential of MSCs is favorable for LG in the native liver scaffold, providing a reliable means to engineer these grafts for use in the clinical setting. Their results suggest that administration of MSCs into the decellularized liver scaffold enriched the microenvironment for factors related to LG and they served as supportive cells for hepatocyte maintenance and protein production [103].

BM-MSCs have been reported to have a beneficial effect on other cell types in cocultures [104, 105], including hepatocytes [106].

#### 3.3.11. Evidence from MSCs Combined with Scaffolds and LG

Herein, role of MSCs in association with scaffold in the enhancement of LG is described. Literature suggests that the significance of liver ECM (extracellular matrix) for differentiation of MSCs has been recognized recently. The lack of ECM cues in a healthy liver resulted in a failure to trigger the differentiation of MSCs. In accordance with this, it was shown that BM-MSCs can differentiate into HLCs (hepatocyte-like cells) in the presence of either damaged liver-conditioned medium or medium supplemented with serum from individuals with liver damage [107].

The pretreatment of MSCs with injured liver tissue represents a novel strategy to augment the differentiation ability of cells towards hepatic lineages. Furthermore, the pretreated MSCs demonstrated better survival, proliferation, differentiation, and functional abilities [92].

Researchers have investigated MSCs for fine-tuned transdifferentiation towards hepatic lineages, with a defined milieu of growth factors and chemicals in a sequential manner, in association with extracellular matrix (ECM) scaffolds [108]. Growth factors in combination with ECM enhance the attachment, proliferation, and differentiation of stem cells, thus mimicking the in vivo microenvironment [109].

Most of the studies investigating MSC differentiation to hepatocytes have been performed on monolayer cultures. MSCs, due to their easy accessibility and possible transdifferentiation potential towards hepatocytes, serve as a prospective cell source for liver tissue engineering [110]. However, such methodologies result in inefficient and heterogenous differentiation, as the two-dimensional (2D) cultures do not mimic the natural liver microenvironment. Three-dimensional (3D) culture systems support an effective and homogenous differentiation by providing structural integrity, promoting improved cell-cell and cell-ECM interactions due to high surface area and porosity thus ensuring growing hepatic tissue remodelling. Evidence suggests that hepatocytes, because of their anchorage dependence, proliferate better on 3D nanobrous scaffolds and form functional aggregates [111, 112].

An ideal strategy for stem cells and nanotechnology based liver tissue engineering would involve suitable biocompatible scaffolds, an effective stem cell source, and a hepatomimetic microenvironment [113, 114].

Proofs of concept are published studies from different researchers. Piryaei et al. [91] have successfully differentiated mouse BM-MSCs into hepatocyte-like cells on ultraweb nanobers.

Similarly, Kazemnejad et al. [115] could derive functional hepatocyte-like cells from human BM-MSCs on a biocompatible three-dimensional nanofibrous scaffold.

Furthermore, Van Poll et al. [11] provided the first clear evidence that delivery of MSC-CM (CM: cellular matrix) can dramatically reduce cell death and enhance LG in D-galactosamine-induced fulminant hepatic failure in vivo and in vitro. These authors reported that MSC-CM therapy led to a 90% reduction in apoptotic hepatocytes and a threefold increase in the number of proliferating hepatocytes in vivo. In addition, the authors demonstrated that secretions from MSCs have a direct inhibitory effect on hepatocyte death and a stimulatory effect on proliferation in ex vivo assays [11].

#### 3.3.12. Engraftment and Hepatocyte Differentiation of hMSC Transplant

In very important study by di Bonzo et al., authors examined whether intravenously transplanted (caudal vein) highly purified hMSCs, obtained from bone marrow donors and expanded ex vivo, were able to engraft in normal as well as injured liver of nonobese diabetic severe combined immunodeficiency (NOD-SCID) mice and to show detectable signs of differentiation into cells of hepatic lineage [116].

Their results prove that although liver engraftment of cells of human origin was unequivocally more prominent under conditions of chronic injury (induced by chronic intraperitoneal injection of CCl4), data from both normal liver and acute liver injury (induced by single intraperitoneal injection of CCl4) indicate definitely that in vivo differentiation of intravenously transplanted hMSCs into hepatocyte-like cells represents a relatively rare and quantitatively unsatisfactory event. This is clearly documented by the very few human cells with hepatocyte-like morphology (ranging from less than 0.1% to 0.23%) and the very low or undetectable levels of human transcripts for *α*-FP, CK-18, CK-19, and albumin in chimeric livers. As a consequence, researchers of this paper proved that transplanted hMSCs have the potential to migrate into normal and injured liver parenchyma, particularly under conditions of chronic injury, but differentiation into hepatocyte-like cells is an exceptional event [116].

Only two and perhaps inconclusive in vivo studies have investigated the hepatic “fate” of transplanted MSCs. In the first study [117], hMSCs were directly injected into injured liver parenchyma of immunosuppressed rats and engraftment was rather obviously limited to the site of injection, with an estimated differentiation efficiency of 0.5% at 4 weeks after transplantation, the standard time point in most studies.

In a second study, human adipose stem cells, rather than hMSCs, were transplanted intravenously into NOD/SCID mice [118], but engraftment/differentiation, perhaps again very limited, was documented only by morphological detection of human albumin and the analysis was not extended later than 10 days after transplantation.

Human MSCs transplantation either by direct injection or by the intravenous route is unlikely to deposit them into the liver-specific stem cell niche [119, 120].

Another interesting research by Popp et al. [121] suggested that multipotent adult BM-MSCs are a cell population that can be expanded to large numbers in culture. BM-MSCs might be differentiated towards hepatocytes in vitro and, thus, are promising candidates for therapeutic applications in vivo. In this study, researchers compared the efficacy of BM-MSCs versus hepatocytes to contribute to LG in a rat model of prolonged toxic hepatic injury. Liver damage was induced by injection of CCl4 or allyl alcohol with and without retrorsine pretreatment [121].

Their results indicate that syngeneic adult BM-MSCs have no contribution to LG in a rat model of prolonged hepatic injury, applying various proliferation stimuli, whereas hepatocytes effectively engraft and proliferate under similar conditions. Authors of this study demonstrated that hepatocytes readily engraft and proliferate in recipient livers after transplantation. However, BM-MSCs cell engraftment could not be observed under similar conditions. This indicates that differentiation of BM-MSCs into hepatocytes does not occur during LG, at least, not to an extent that is of potential clinical benefit. In contrast to MSCs (which can be easily obtained and be propagated in culture), hepatocytes (which cannot be easily obtained and cannot be cultivated long-term) turned out to be the more effective cell population for liver-directed cell therapy [121]. Overall, the clinical implementation of liver stem cell therapy remains promising [122] but ambiguous so far.

Consequently, findings of Popp et al. [121] demonstrate no contribution of multipotent MSCs to LG in a rat model of prolonged hepatic injury.

#### 3.3.13. Profibrogenic Potential of hMSC Transplant

Evidence from murine models involving bone marrow ablation shows that transplanted bone marrow cells were able to engraft the liver from bone marrow during liver diseases and to significantly contribute to liver fibrosis by differentiating into profibrogenic myofibroblast-like cells, with hepatocyte-like transdifferentiation being again a rare event [98]. In particular, these authors provided convincing evidence indicating that the predominant source of myofibroblasts in their models was represented by MSCs [98].

Respectively, another research by Baertschiger et al. [123] demonstrated that, after intrahepatic injection of human BM-MSCs into a mouse model of acute liver injury induced by 2/3 hepatectomy, MSCs expressed alpha smooth muscle actin and merged with collagen deposition suggesting that MSCs are able to exhibit a fibrogenic phenotype.

In accordance with these findings, the study of di Bonzo et al. [116] conceptually confirm and complete data obtained in the murine models [98]. Particularly, results of di Bonzo et al. [116] prove that MSCs adopted a myofibroblast-like shape after transplantation in a model of acute liver injury. This fibrogenic potential may be related to the high expansion of MSCs; it is possible that some clones are prone to differentiate into fibroblast-like cells and thus contribute to the fibrotic process of the injured liver. Subsequently, in the situation of acute liver injury, MSC transplantation might lead to unwanted effects and contribute to the fibrotic reaction. The detection of MSCs expressing alpha smooth muscle actin further leads authors of this paper to point out a possible fibrogenic potential of MSCs.

Authors suggest that, in chronologically injured chimeric livers a significant number of MSCs are positive for human HLA-I antigens and/or GFAP. Furthermore, MSCs exhibit morphological features of myofibroblasts. This last fact supports the view that MSCs recruited from bone marrow to chronologically injured liver may display a profibrogenic potential. As a consequence, this study [116] demonstrates that hMSCs transplant has a profibrogenic potential, which should be not underevaluated. Accordingly, further studies are needed to elucidate whether MSCs may behave as potentially “useful” or “dangerous” cells in the scenario of chronic liver diseases [116].

### 3.4. Second Section

#### 3.4.1. Outcomes from MSCs Transplantation following PH

For the aims of this systematic review 7 studies (*n* = 7) were included and reviewed in regard to MSCs transplantation following PH [14, 16–21]. From these studies, in five, authors performed ADSCs transplantation [14, 17–19, 21], whereas, in two, authors transplanted BM-MSCs [16, 20] (Table 1). In all included papers on MSCs transplantation following PH, authors used rats as experiment animals, except from the study of Saito et al. [19], in which authors have performed their experiments in mice. In this part all included studies which respond to the primary endpoints of this systematic review and, consequently, outcomes from MSCs transplantation following PH will be reviewed. Results from ADSCs and BM-MSCs transplantation will be reviewed separately in results specific parts 1 and 2, respectively (Table 1).

*(a) Results from Studies on ADSCs Transplantation after PH*. In five studies authors performed ADSCs transplantation following PH [14, 17–19, 21]. Among included literature on MSCs transplantation, specifically reviewed for the aims of this systematic review, effects of MSCs transplantation in the enhancement of LG after PH and improvement of the hepatic function are evaluated and compared. These are the primary endpoints of this study.

Among included studies, Seki et al. conducted ALI either by hepatectomy or through temporary clamping of hepatoduodenal ligament to reproduce an ischemia/reperfusion model [17]. ADSCs were administered through the penile vein, and the LG rate was found to be higher compared to the sham and the control group. The authors of this study prove that LG rate after ALI is higher in the group which received ADSCs, which highlights the beneficial role of ADSCs transplantation in the enhancement of LG. Furthermore, findings of Seki et al. [17] highlight that, even in the peripheral portion of the liver that can be observed by intravital microscopy, stasis of ADSCs was observed in the periportal areas after injection of ADSCs into the femoral vein. It was speculated that more ADSCs may stay in the central portion of the liver. Based on these findings, ADSCs may provide beneficial effects towards the process of LG in the periportal area in a paracrine fashion. Further investigation is necessary to clarify the precise mechanism of ADSC-mediated liver proliferation. In the same study, authors observed that the level of mRNA and protein expression of many of these factors was significantly enhanced 1 day after hepatectomy in the ADSCs group compared with the sham group. They observed that these changes on day 1 reflect the data obtained on LG rate and mitotic index on day 2. This study demonstrates that ADSC transplantation promotes LG, which is associated with the upregulation of hepatic regeneration-associated factors after hepatic ischemia-reperfusion and subsequent hepatectomy. Authors claim that ADSC transplantation may have therapeutic potential for cases of major hepatectomy with repeated hepatic ischemia-reperfusion [17].

In the study of Sun et al. [18], authors utilized serum from rats subjected to 70% PH and investigated the differentiation ability of rat ADSCs in vitro 24 h after PH. In this study, stem cells transformation was recorded while the intensity of liver injury was improved. Furthermore, the potential role of ADSCs in vivo, following PH injury, was also explored. The authors found that ADSCs treated with serum from rats, differentiated into hepatocyte-like cells, expressed *α*-FP, secreted albumin, synthesized urea, and acquired cytochrome P450 activity. Moreover, ADSCs upregulated the expression of IL-6 and HGF transiently in vitro, although the hepatic differentiation efficiency was extremely low. The results of this study demonstrate that the administration of ADSCs ameliorated liver injury and promoted LG. Nevertheless, ADSCs in vivo, after 24 h of 70% PH, do not increase IL-6 and HGF expression. These results prove that ADSCs adequately display a multidimensional role, in order to reduce complex hepatic injuries that lead to irreversible organ failure [18].

Saito et al. [19] highlight the beneficial role of ADSCs transplantation after 70% PH and ischemia/reperfusion, in mice. Particularly, authors demonstrated that, in vivo, ADSCs had beneficial effects for liver injury after subsequent hepatectomy and ischemia/reperfusion and, in vitro, ADSCs' trophic molecules, including HGF and VEGF, protected hepatocytes. However, authors found that suppression of VEGF by the administration of bevacizumab did not affect the protective effects of ADSCs. In the same paper, authors demonstrated that a variety of bioactive cytokines secreted by transplanted ADSCs, such as HGF, FGF, and VEGF, might be involved in restoring liver function and promoting regeneration [19].

Koellensperger et al. [21] utilized ADSCs and injected them directly to the hepatic parenchyma. Experiment animals were Sprague Dawley rats that have undergone a 2/3 PH accompanied by chemical-induced liver injury by retrosine and allyl alcohol. The authors found an increase in albumin levels in the postoperative period. The authors of this paper also identified ADSCs until 12 weeks after transplantation [21].

In a recent study of good quality published by Tautenhahn et al. [14], authors performed a 90% PH in DPPIV-deficient F344-Fischer rats and transplanted ADSCs by splenic application. It is noteworthy that authors of this article performed the most extensive PH described in included published literature. In this paper, authors transplanted ADSCs that were predifferentiated in vitro into hepatocytic cells. Authors found that ADSCs transplantation supported survival after PH and decreased liver-related blood parameters indicative for the improvement of liver function. Furthermore, ADSCs decreased the apoptotic rate and increased the proliferation rate. Nevertheless, ADSCs integration into the host liver was not possible and the explanation for this is the fact that the experimental time period of 48 hours was considered too short. Administered ADSCs ameliorated hepatic dysfunction and improved LG after extended resection by paracrine mechanisms. The authors of this study claim that MSCs may represent a new therapeutic option to treat posthepatectomy acute liver failure [14].

*(b) Results from Studies on BM-MSCs Transplantation after PH*. Among included and reviewed studies for the aims of this article, only two studies were retrieved in which authors transplanted BM-MSCs, following PH in rats [16, 20].

In the first study, Arikura et al. [16] immediately injected normal BM-MSCs through the portal vein after 70% PH in congenic Nagase's albumin-deficient rats. Four weeks after the injections of BM-MSCs, authors found an expression of albumin mRNA and protein, which was observed in the liver of the albumin-deficient rats, used in the experiment protocol. Moreover, albumin was present in serum. This study suggests the beneficial role of BM-MSCs transplantation after PH.

Meanwhile, Li et al. [20] transplanted BM-MSCs in rats after a 70% PH. The route of administration, adopted in their experiments, was the portal vein or the tail vein. Authors found that BM-MSCs could differentiate into hepatocytes after severe liver injury. Furthermore, authors demonstrated that the injected BM-MSCs could migrate to the damaged liver and might differentiate into hepatocytes to promote LG after PH in rats. Autologous stem cells might provide a promising therapeutic effect on LG after surgery or liver injury [20].

#### 3.4.2. Outcomes from ADSCs and BM-MSCs Transplantation in Models of Liver Fibrosis and Chronic Liver Injury

For the aims of this systematic review, 6 studies were included and reviewed in regard to MSCs transplantation in models of acute and chronic liver failure, liver fibrosis, and liver cirrhosis [22–27]. From these studies, in five, authors performed ADSCs transplantation [23–27], whereas only in one study did authors transplant BM-MSCs [22] (Table 2). Among included studies which fulfilled secondary endpoints of this study, in three articles [22, 24, 25] authors used rats for their experiments, while, in the remaining three studies [23, 26, 27], authors performed their experiments in mice. It is noteworthy that, in 2 included studies [23, 27], authors used ADSCs of human origin. Nevertheless, the human origin of ADSCs was not limiting factor for inclusion, because it is not an exclusion criterion, according to the strict methodological design of this article (Figure 1).

In this part all included studies which respond to the secondary endpoints of this systematic review are reviewed. Consequently, herein, are reviewed studies on MSCs transplantation in models of acute and chronic liver failure, liver fibrosis, and liver cirrhosis. Results from ADSCs and BM-MSCs transplantation will be reviewed separately in results specific parts 1 and 2, respectively (Table 2).

*(a) Results from Studies on ADSCs Transplantation*. Among considered studies, which fulfilled the secondary endpoints of this article, in five studies authors performed ADSCs transplantation [23–27].

Particularly, in the study of Harn et al. [24], authors transplanted ADSCs with direct liver injection, in rats. Authors found that the transplanted ADSCs were differentiated into albumin and *α*-FP secreting liver-like cells, one week after transplantation. This fact demonstrates the differentiation ability of ADSCs in hepatocytic cells and their incorporation in the liver parenchyma, after administration. Furthermore, authors demonstrated that liver function recovered significantly after ADSCs transplantation, as determined by biochemical analyses that analyzed total bilirubin, PT, and albumin levels. This study shows that ADSCs limit severity of chronic hepatic failure and increase survival [24].

In the study of Okura et al. [23], authors performed their experiments in NOD-SCID mouse (nonobese diabetic severe combined immunodeficiency mouse) and transplanted ADSCs of human origin. The authors describe a new method for generation of functional hepatocyte-like cell clusters, using floating culture. They found that induced functional hepatocyte-like cell clusters functioned effectively both in vitro and in vivo and this is a finding of crucial importance. They demonstrated that generated hepatocyte-like cell clusters were transplanted into NOD-SCID mouse with chronic liver injury and resulted in a significant improvement of serum albumin and total bilirubin levels. This study proves that ADSCs limit severity of chronic hepatic failure and increase survival.

With the two aforementioned studies of Harn et al. and Okura et al., an important conclusion is deduced; ADSCs can limit severity of chronic hepatic failure as well as increase survival rate [23, 24].

In the study of Wang et al. [25], authors administered ADSCs in the portal vein or in the tail vain of rats and assessed their effects in a model of CCl4-induced liver fibrosis. Authors found increased portal vein perfusion and microcirculation improvement. ADSCs transplantation resulted in fibrosis decrease, which suggests the beneficial role of ADSCs transplantation in the treatment of liver fibrosis [25].

In the study of Seki et al. [26] authors transplanted ADSCs directly to the hepatic parenchyma of mice, to assess the effects of ADSCs in a cirrhosis model, induced by nonalcoholic steatohepatitis. Authors found that ADSCs transplantation provoked downregulation of inflammatory cells. Furthermore, ADSCs favored LG by upregulation of regenerative genes.

In the study of Saidi et al. [27], authors administered ADSCs of human origin in a model of CCl4-induced acute liver failure in mice. Mice were treated with human ADSCs before CCl4-induced acute liver failure. Authors found that ADSCs attenuated liver injury and improved survival. This study shows that ADSCs transplanted in a model of acute liver failure may offer benefits in survival of mice and this is an important outcome, particularly if shifted to the clinical setting.

*(b) Results from Studies on BM-MSCs Transplantation*. Only in one of the reviewed studies, authors administered BM-MSCs. In the study of Abdel Aziz et al. [22], authors isolated CD29+ MSCs from the bone marrow of males and injected them into the tail vein, in a female rat fibrosis model. Authors found that BM-MSCs could differentiate into hepatocyte-like cells and reduce fibrosis by decreasing the precipitation of collagen. These results support the role of MSCs as a therapeutic agent for liver disease [22].

## 4. Discussion

The results of this systematic review suggest that MSCs are multipotent and self-renewing cells which exhibit profitable immunomodulatory properties. MSCs attenuate the immune and inflammatory responses by the secretion of cytokines [9, 10, 28]. These features enhanced LG in acute and chronic liver pathologies in rodents. Furthermore, MSCs ameliorated LG by presenting proproliferative and antiapoptotic properties, thus hampering disease progress [11–13]. These proregenerative properties of MSCs may enhance the regenerative capacity of the remaining liver after extensive resection [14].

In this article, the role of MSCs in the enhancement of LG and in the improvement of liver function after PH was assessed. Furthermore, this study evaluated the effects of MSCs transplantation as treatment option for acute and chronic liver injury. Included studies assessed effects of ADSCs and BM-MSCs transplantation. All literature suggests that ADSCs may be considered more profitable in comparison with BM-MSCs, because ADSCs are abundant and can be harvested with minimally invasive procedures [41]. In addition, it has been reported that ADSCs produce more bioactive factors than do BM-MSCs [19, 43].

MSCs transplantation was proved to have important benefits after PH or in acute and chronic liver injury. For all these reasons, the results of this systematic review are encouraging and highlight the urgent need for more experimental and clinical studies on MSCs transplantation after PH to prevent posthepatectomy liver failure [14, 15] and improve liver function, obtaining higher LG rates [23–25].

ADSCs are committed at higher rates in hepatic lineage (compared to BM-MSCs) after pretreatment with growth factors in order to differentiate into hepatic-like cells [13, 54, 55]. Moreover, it is noteworthy that ADSCs show higher rates of proliferation as well as stronger commitment to hepatic lineage compared to BM-MSCs [56, 58].

The results of included reviewed studies for the aims of this systematic review proved that MSCs transplantation is an innovative means to promote LG and ameliorate liver function following PH, as suggested by different published studies [14, 16–21]. Furthermore, MSCs have beneficial effects in the treatment of acute and chronic liver failure, liver fibrosis, and liver cirrhosis [22–27].

In this systematic review, ADSCs and BM-MSCs were considered and compared. ADSCs were proved to be more profitable than BM-MSCs considering their easier isolation. Findings from assessed studies have certain shortcomings such as the fact that included studies were experimental studies in rodent models and because they are all single-center experiences.

The originality of this article consists in the fact that this is the first systematic review to assess outcomes from experimental MSCs transplantation following PH in rodents [14, 16–21]. This article also assessed the effects of MSCs in acute and chronic liver injury [22–27]. Furthermore, inclusion and exclusion criteria, methodology of screening, and selection process of included studies are unique in this review.

### 4.1. Assessment of the Outcomes Obtained after MSCs Transplantation

Among PH studies included and reviewed, Arikura et al. [16] performed a 70% PH and consecutively transplanted BM-MSCs directly in the portal vein of albumin-deficient rats, finding albumin production 4 weeks after transplantation. Authors highlight the beneficial role of BM-MSCs in recovery of liver function after PH. On the contrary, Seki et al. [17] administered ADSCs via the penile vein or the femoral vein, performing ischemia/reperfusion, finding higher LG rate than in shame-control groups. Authors claim that ADSCs transplantation promoted LG after PH, particularly in cases with repeated ischemia/reperfusion. Results of Seki et al. [17] are in accordance with Arikura et al. [16] despite the different type of transplanted MSCs. Sun et al. [18] transplanted ADSCs in the tail vein of rats, subjected to a 70% PH and assessed the differentiation ability of ADSCs 24 h after PH. Authors found that ADSCs differentiated into hepatocyte-like cells, expressed *α*-FP, secreted albumin, synthesized urea, and acquired cytochrome P450 activity. Also this study proves the beneficial role of ADSCs which ameliorate liver injury and promote LG. Saito et al. [19] administered ADSCs in mice and found that ADSCs have beneficial and protective effects on liver injury and LG, after a 70% PH and ischemia/reperfusion. Furthermore, the authors demonstrated that HGF and VEGF secreted by ADSCs protect hepatocytes. Suppression of VEGF by bevacizumab administration did not affect the protective effects of ADSCs. Authors demonstrated that ADSCs secrete HGF, VEGF, and FGF which restore liver function and promote LG. Li et al. [20] transplanted BM-MSCs in the portal vein and the tail vein of rats after a 70% PH, finding that injected BM-MSCs can migrate to the damaged liver and differentiate into hepatocytes to promote LG after PH, while Koellensperger et al. [21] transplanted ADSCs directly in the hepatic parenchyma of rats, after 2/3 PH. In this protocol liver injury was chemically induced by retrosine and allyl alcohol. Postoperatively, increased levels of albumin were retrieved and MSCs were identified up to 12 weeks after transplantation. Tautenhahn et al. [14] administered ADSCs by direct splenic application in rats after a 90% PH. This is the only experimental study which adopted this route of administration. In fact, splenic direct application was not adopted in any other study, among the included ones. In this paper, transplanted ADSCs were predifferentiated into hepatocytic cells. Authors proved that ADSCs supported survival following PH, decreased the apoptotic rate, increased the proliferation rate, ameliorated hepatic dysfunction, and improved LG after extended resection by paracrine mechanisms. Furthermore, ADSCs decreased liver-related blood parameters, indicative for the improvement of liver function. Authors claim that ADSCs may represent a new therapeutic option to treat posthepatectomy acute liver failure.

Among the acute and chronic liver failure, liver fibrosis, and liver cirrhosis models [22–27], which assessed the effects of MSCs transplantation, in the study by Okura et al. [23], authors used NOD-SCID mouse and transplanted ADSCs of human origin. Meanwhile, Harn et al. [24] used rats and transplanted ADSCs with direct liver injection. Transplanted ADSCs differentiated into albumin and *α*-FP secreting liver-like cells, 1 week after transplantation. Authors found that ADSCs limit severity of chronic hepatic failure and increase survival. Both Harn and Okura state that ADSCs can limit severity of chronic hepatic failure as well as increase survival rate [23, 24]. Meanwhile, in the study of Wang et al. [25], authors administered ADSCs in the portal vein or in the tail vain of rats and assessed their effects in a model of liver fibrosis. Authors found that ADSCs transplantation resulted in reduction of liver fibrosis. On the contrary, Seki et al. [26] transplanted ADSCs directly to the hepatic parenchyma of mice, in a cirrhosis model, and found that ADSCs favored LG by upregulation of regenerative genes. Meanwhile, Saidi et al. [27] administered ADSCs of human origin in a model of acute liver failure in mice. Also this study proves that ADSCs attenuated liver injury and improved survival, whereas Abdel Aziz et al. [22] used rats and administered BM-MSCs in the tail vein in a rat fibrosis model. Authors found that MSCs could differentiate into hepatocyte-like cells and reduce fibrosis by decreasing precipitation of collagen. All these studies prove the beneficial role of MSCs in preventing posthepatectomy acute liver failure, in improving liver function, and in reducing liver injury parameters and increasing survival.

### 4.2. Critical Appraisal of Included Published Literature on MSCs Transplantation

Among included PH studies [14, 16–21], in four papers, authors performed a 70% PH [16, 18–20], whereas Koellensperger et al. [21] performed a 2/3 PH which is the less extensive PH, among all reviewed studies. On the contrary, Tautenhahn et al. [14] performed the most extensive PH of all included and reviewed literature, for the aims of this review. The percentage of PH does not imply significant differences in attended benefits from MSCs transplantation, although direct comparisons among included studies are methodologically incorrect because all studies have different methodology of reporting and different endpoints and, consecutively, obtained different results.

Furthermore, the route of MSCs administration did not influence obtained results. Portal vein was used as route of administration in two studies [16, 20], whereas penile vein was the preferred administration route only in 1 study [17]. In the same study authors used also femoral vein as administration route [17]. MSCs were transplanted in the tail vein in 2 studies [18, 20]. Direct MSCs transplantation to the hepatic parenchyma was performed in one study [21]. Splenic application of MSCs was performed in one study [14].

Among liver injury studies, MSCs were transplanted in the tail vein in one study [22]. In two studies [24, 26], MSCs were administered directly to the hepatic parenchyma, whereas portal vein and tail vein were the preferential routes of administration in one study [25].

It should be highlighted that this systematic review demonstrates that incomplete reporting in published experimental studies on MSCs transplantation is a significant problem in all published studies under this topic. There is clearly a need for the development of standards of reporting in published literature to permit construction of evidence-based algorithms which will strictly define in which cases MSCs transplantation should be proposed and considered after PH, to prevent liver failure, also in the clinical setting. In order to permit a broad adoption of MSCs transplantation following PH in the clinical routine, to avoid acute liver failure, more experimental studies are needed and, especially, further systematic reviews and meta-analyses. Only with generalizable results from well conducted experimental studies, MSCs transplantation could shift from the experimental to the clinical setting.

The attained benefits are many, although an extensive body of high quality of literature is still needed, to avoid shortcomings of current studies which influence negatively generalizability of the results, in absence of other systematic reviews or meta-analyses, under this topic.

## 5. Conclusions

MSCs are multipotent and self-renewing cells which possess a broad therapeutic potential and have been evaluated for their in vivo, in vitro immunomodulatory and “tissue reconstruction” properties, which could make them appealing in various clinical applications and particularly in organ transplantation [28].

This systematic review demonstrates that MSCs transplantation has beneficial effects because it promotes LG, improves liver function, and increases survival after PH and acute or chronic liver injury. MSCs transplantation represents a new concept to prevent posthepatectomy acute liver failure following extensive hepatectomy [14]. Published literature proved that MSCs transplantation is associated with the upregulation of hepatic regeneration-associated factors [26]. Furthermore, MSCs may differentiate into hepatocyte-like cells and protect hepatocytes by paracrine mechanisms [19]. This article included 13 studies on MSCs transplantation. Seven studies responded at the primary endpoints and 6 at the minor endpoints of this review.

ADSCs were proved to be more profitable than BM-MSCs in the included studies, considering the easier isolation with minimally invasive procedures [41], the higher commitment rate, and the stronger secretion of bioactive factors. ADSCs show higher differentiation ability in hepatocytes than do BM-MSCs [19].

This systematic review was designed to include only experimental studies in rodent models. Included literature has certain methodological shortcomings. All studies are single-center experiences. Different methodological design of included studies and different endpoints make direct comparison of obtained results methodologically incorrect. More experimental and clinical studies are needed to generalize results and permit safer clinical application of MSCs in routine in liver disease and other human pathologies.

It is certain that, in the next years, many other applications of MSCs transplantation will broaden the spectrum of therapeutic applications and will transit out of the frontiers of liver disease. More experimental and clinical trials are needed. High level of evidence is required to permit obtainment of more generalizable results. Consecutively, further systematic reviews and meta-analyses will permit obtainment of more generalizable conclusions. Well designed clinical trials will permit transition of MSCs transplantation to the clinic, for liver disease.

## Figures and Tables

**Table 1:** Specific data of included studies on MSCs transplantation after PH, type of MSCs, route of transplantation, and obtained results (chronological order of publication). In this table, results from major endpoints of this study are presented.

Study	Animal model	Type of MSCs	Route of administration	Results
Arikura et al. (2004) [16]	Rats	BM-MSCs	Portal vein	(a) BM-MSCs from normal rats were infused via the PV into the livers of congenic Nagase's analbuminemic rats immediately after 70% PH (b) BM-MSCs transplantation in albumin-deficient rats resulted in albumin production 4 wks after transplantation

Seki et al. (2012) [17]	Rats	ADSCs	(a) Penile vein (b) Femoral vein	(a) In I/R model higher LG rate was found than in shame/control groups (b) Stasis of ADSCs in the periportal areas after injection of ADSCs but more in the central portion of the liver (c) ADSCs' transplantation promotes LG after PH, particularly in cases with repeated I/R

Sun et al. (2013) [18]	Rats	ADSCs	Tail vein	(a) The effects of serum from rats subjected to 70% PH on the differentiation ability of rat ADSCs in vitro were investigated, 24 h after PH (b) The potential role of ADSCs in vivo following PH injury was also explored (c) ADSCs were treated with serum from rats, differentiated into hepatocyte-like cells, expressed *α*-FP, secreted alb, synthesized urea, acquired cytochrome P450 activity, and upregulated the expression of IL-6 and HGF transiently in vitro, although the hepatic differentiation efficiency was extremely low (d) Using ADSCs, liver injury was ameliorated and LG was promoted (e) ADSCs, in vivo, after 24 h of 70% PH do not increase IL-6 and HGF expression

Saito et al. (2013) [19]	Mice	ADSCs	NR	(a) ADSCs have beneficial protective effects on liver injury and LG, after 70% PH and I/R (b) VEGF and HGF secreted by ADSCs protect hepatocytes (c) Suppression of VEGF by bevacizumab administration did not affect the protective effects of ADSCs (d) ADSCs secrete HGF, VEGF, and FGF which restore liver function and promote LG

Li et al. (2013) [20]	Rats	BM-MSCs	(a) Portal vein (b) Tail vein	(a) Rats received BM-MSCs through portal vein or tail vein, after a 70% PH (b) Injected BM-MSCs can migrate to the damaged liver and differentiate into hepatocytes to promote LG after PH

Koellensperger et al. (2013) [21]	Rats	ADSCs	Hepatic parenchyma	(a) Rats received ADSCs with injection directly to the hepatic parenchyma, after 2/3 PH, chemically induced liver injury by retrosine and allyl alcohol (b) Postoperatively, increased levels of albumin were retrieved (c) MSCs were identified up to 12 weeks after transplantation

Tautenhahn et al. (2016) [14]	Rats	ADSCs	Splenic application	(a) Rats received ADSCs by splenic application following 90% PH (b) Transplanted ADSCs were predifferentiated into hepatocytic cells (c) ADSCs supported survival after PH (d) ADSCs decreased liver-related blood parameters indicative for the improvement of liver function (e) ADSCs decreased the apoptotic rate and increased the proliferation rate (f) The experimental time period of 48 hours was too short to allow for integration of MSCs into the host liver (g) MSCs ameliorated hepatic dysfunction and improved LG after extended resection by paracrine mechanisms (h) They may represent a new therapeutic option to treat posthepatectomy acute liver failure

NOD-SCID mouse: nonobese diabetic severe combined immunodeficiency mouse.

I/R: ischemia/reperfusion.

**Table 2:** Specific data of included studies on MSCs transplantation in acute and chronic liver failure, liver fibrosis and liver cirrhosis models, type of MSCs, route of transplantation, and obtained results (chronological order of publication). In this table, results from minor endpoints of this study are presented.

Study	Animal model	Type of MSCs	Route of administration	Results
Abdel Aziz et al. (2007) [22]	Rats	BM-MSCs	Tail vein	(a) Isolated CD29+ MSCs from the BM of males and injected them into the tail vein in a female rat fibrosis model. (b) The MSCs could differentiate into hepatocyte-like cells and reduce fibrosis by decreasing precipitation of collagen.

Okura et al. (2010) [23]	NOD-SCID mice	ADSCs (To note that in this study, the authors used ADSCs of human origin)	NR	(a) A new method for generation of functional hepatocyte-like cell clusters is described, using floating culture. Induced functional hepatocyte-like cell clusters functioned effectively both in vitro & in vivo. (b) The generated hepatocyte-like cell clusters were transplanted into NOD-SCID mouse with chronic liver injury, resulting in a significant improvement of serum alb & total brb levels. (c) ADSCs limit severity of chronic hepatic failure and increase survival.

Harn et al. (2012) [24]	Rats	ADSCs	Direct liver injection	(a) The transplanted ADSCs differentiated into albumin & *α*-FP secreting liver-like cells, 1 week after transplantation. (b) Liver function recovered significantly, as determined by biochemical analyses that analyzed total brb, PT & albumin levels (c) ADSCs limit severity of chronic hepatic failure & increase survival.

Wang et al. (2012) [25]	Rats	ADSCs	(a) Portal vein or (b) Tail vein	(a) In a model of CCl4-induced liver fibrosis, authors administered ADSCs. (b) Findings: increased portal vein perfusion and microcirculation improvement. (c) Result: decreased fibrosis.

Seki et al. (2013) [26]	Mice	ADSCs	Directly to hepatic parenchyma	(a) Model of cirrhosis due to nonalcoholic steatohepatitis. (b) ADSCs transplantation provoked downregulation of inflammatory cells. (c) ADSCs favored liver regeneration by upregulation of regenerative genes.

Saidi et al. (2015) [27]	Mice	ADSCs (to note that, in this study, the authors used ADSCs of human origin)	NR	(a) Model of CCl4-induced acute liver failure. (b) Mice were treated with human ADSCs prior CCl4 induced acute liver failure. (c) The results prove that ADSCs attenuated liver injury and improved survival.

NOD-SCID mouse: nonobese diabetic severe combined immunodeficiency mouse.

**Table 3:** Advantages and disadvantages of transplantation of ADSCs compared to BM-MSCs and minimal criteria to define MSCs are reported.

BM-MSCs		ADSCs		Minimal criteria to define MSCS
Advantages	Disadvantages	Advantages	Disadvantages	
Earliest found	Donor shorted	Abundant	NR	*Expression of * CD105+ CD73+ CD90+ *Lack of expression of*: CD45− CD34− CD14− or CD11b− CD79a− or CD19− HLA-DR (−)

Largest reserved	Age related	Less Invasive	NR	Adhesion to plastic in standard culture conditions

Prevalently used	Highly invasive	Easily isolated	NR	In vitro differentiation into osteoblasts, adipocytes and chondroblasts

	Easily rejected	Immunosuppressive	NR	

## Abbreviations

MSCs: Mesenchymal stem cells
ADSCs: Adipose tissue-derived mesenchymal stem cells
BM-MSCs: Bone marrow-derived mesenchymal stem cells
LG: Liver regeneration
ALI: Acute liver injury
PH: Partial hepatectomy.
